# Improvement of Anchorage Performance of Carbon Fiber-Reinforced Polymer Cables

**DOI:** 10.3390/polym14061239

**Published:** 2022-03-18

**Authors:** Tae-Kyun Kim, Woo-Tai Jung

**Affiliations:** Department of Structural Engineering Research, Korea Institute of Civil Engineering and Building Technology, 283, Goyang-daero, Ilsanseo-gu, Goyang-si 10223, Gyeonggi-do, Korea; woody@kict.re.kr

**Keywords:** fiber-reinforced polymer, CFRP cable, multi-anchorage system, compression sleeve, prestressed concrete

## Abstract

Prestressed concrete composed of steel materials is increasingly used in various social infrastructures, such as bridges (cables), nuclear containment structures, liquefied natural gas (LNG) tanks, and structural reinforcements. This study aimed to substitute the steel in bridge cables with fiber-reinforced polymers (FRPs) to prevent the damage caused by the performance degradation of corroded prestressed steel. An optimized single-anchorage system was derived by applying multiple variables, such as the surface treatment, number of insert layers, and sleeve processing companies, to improve the maximum load and bonding with the anchorage system sleeve using the carbon FRP (CFRP) cable. The B-L-4 specimen (sleeve specifications of company B, longitudinal surface treatment, and four insert layers) was determined to be the optimized single-anchorage system. When the tensile test was conducted after applying the optimized single-anchorage system to the three- and seven-multi-anchorage systems, the tensile performances of B-L-4 were 100 and 95% of the one-multi-anchorage system, respectively. Considering that the problems associated with the construction of three- and seven-multi-anchorage systems have been addressed, these systems can be applied to actual bridges in the future, and can significantly benefit their maintenance.

## 1. Introduction

Prestressed concrete (PSC) is commonly used in various social infrastructures, including bridges (cables), nuclear containment structures, liquefied natural gas (LNG) tanks, and structural reinforcements [1]. Among the members of PSC structures, PS steel and anchorage systems are essential and require proper construction for their accurate performance. The anchorage systems secure the PS steel in position, while the PS steel applies force on the concrete members [2,3]. Different types of PS steel, such as PS steel wires, bars, PS strands, deformed PS steel wires and bars, and other PS materials, are primarily used in bridges [2,3]. Typically, PS steel exhibits specific properties, including low relaxation, high tensile strength, high yield ratio, moderate ductility and toughness, high resistance to stress corrosion, high bonding strength with concrete, appropriate fatigue strength, and linearity [2,3]. However, the quality and performance of PS steel naturally decreases due to corrosion over time [4,5]. Different anchorage methods used for PS steel include wedge-, pressure- (rivet head and nut types), and loop-types. The wedge-type anchorage secures the PS steel using a wedge action based on the friction force between the PS steel and the anchorage system [6,7,8] and is primarily used in the Freyssinet, VSL, and CCL methods to anchor PS steel wires and strands. The pressure-type anchorage is divided into rivet head and nut-type methods, wherein the ends of the PS steel wires and rods processed as nail heads and screw threads inserted into nuts, respectively, are supported using pressure plates [9,10,11]. The typical method of the rivet head type is BBRV, while that of the nut type includes the Dywidag, Lee–McCall, and stress steel methods [12,13,14]. The loop-type is an anchorage method based on external bonding with concrete or pressure, in which the PS steel wires and strands processed in a loop shape are embedded in the concrete. The Leoba and Baur–Leonhardt methods represent loop-type anchorages. Anchorage systems have also been applied using various other methods [15,16,17]. However, the performance degradation of PS steel due to corrosion affects the quality of these structures. Many previous studies have focused on lecture line compression sleeves, comprising steel strands. Although cables and anchorage systems composed of fiber-reinforced polymers (FRPs) have been investigated as an alternative, their research remains scarce to date [18,19,20,21]. Yang et al. (2020) analyzed the tensile and shear characteristics of concrete using glass FRP (GFRP), aramid FRP (AFRP), basalt FRP, and basalt and carbon FRP cables; they also analyzed the performance of the concrete after applying the wedge-type anchorage system. Damiani et al. (2021) investigated performance considering the angle of the wedge system using FRP cables, while Wang et al. (2018) examined the split-wedge, curved-wedge, and non-metal wedge anchorage systems using CFRP cables. Zhuge (2010) also evaluated the stress of a conical barrel based on the mechanical behavior of the wedge-type anchor for CFRP tendons. Campbell et al. (2000) observed the performance and changes in the wedge for individual and cyclic loads based on the wedge-type stainless steel anchorage system using FRP tendons [22,23,24,25,26]. Thus, most existing studies have focused on the wedge-type anchorage system using steel strands [27,28,29,30]. In this study, we used a pressure-type anchorage system based on a hollow metal sleeve, investigated FRPs as substitutes for steel in bridge cables, and developed an optimized anchorage system for FRPs. Among the various FRPs, we selected CFRPs for our investigation. Compared to conventional steel materials, FRPs as composite materials exhibit certain beneficial aspects, such as light weight, high specific strength, high specific stiffness, weather resistance, non-magnetic properties, low thermal conductivity, and high durability. In particular, they are resistant to corrosion, while PS steel can result in human casualties and property damage owing to fractures caused by corrosion. However, a major drawback of FRPs is their brittle failure tendency.

Thus, to improve the pressure performance of FRPs, we performed an experiment using an anchorage system based on a sleeve and CFRP cables. The previous studies employed processed CFRP forms and the steel strand method. However, in the present study, we experimentally derived the optimized single-anchorage system by applying multiple variables, such as surface treatment according to the CFRP cable direction, the number of insert layers, and sleeve processing companies. After applying the single-anchorage system to three- and seven-multi-anchorage systems, the tensile tests were conducted to compare and analyze the performance. The obtained results can serve as a database for developing optimized cables, such as CFRP strands and anchorage systems. Thus, FRPs can reduce the damage caused by the corrosion of PS steel, and ensure significant efficiency in maintaining bridges when applied to actual bridges.

## 2. Materials and Methods

### 2.1. CFRP

#### 2.1.1. Carbon Fibers

Carbon fibers originated when Thomas Edison carbonized bamboo fibers and used them as filaments for a light bulb in the 1880s. However, the filaments were fragile and significantly different from those used currently. Nevertheless, the origin of the present-day high-performance carbon fibers can be attributed to the rayon-based carbon fibers developed in the mid-1950s [31]. Although carbon fibers are commonly referred to as graphite fibers without distinction, those heat-treated at a temperature of 1700 °C or higher are graphite fibers. In contrast, fibers heat-treated at lower temperatures are carbon fibers. However, these two fiber types are generally not distinguished, as graphite fibers are also composed of carbon atoms [32].

Table 1 shows the characteristics of FRP fibers. Materials required for manufacturing carbon fiber products are referred to as precursors. Typically, carbon fibers are classified into rayon-, polyacrylonitrile (PAN)-, and pitch-based carbon fibers, with respect to the precursor type. The first commercial carbon fiber developed in the mid-1950s used rayon as a precursor, and these rayon-based carbon fibers were initially dominant [31]. However, after successful manufacturing of PAN-based carbon fibers in Japan in 1960, they have been commonly used as composite materials. In 1965, carbon fibers were manufactured using a refined petroleum by-product, known as pitch, as a precursor. Nevertheless, PAN-based carbon fibers are predominantly used as composite materials. However, the price of carbon fibers can be reduced and their applications expanded considering that the process of manufacturing pitch-based carbon fibers has become common [32].

#### 2.1.2. Binder

The binder of a composite material forms structural geometry by maintaining the fixed fibers in position, which is also referred to as a matrix. Considering that the reinforcing fibers are resistant to the load, the binder transfers the stress between the fibers and protects them from the external environment [33]. Although the binder barely contributes to the axial load of the reinforcing fibers, it primarily resists the shear load because the fibers lack excellent shear properties. Furthermore, the binder is essential for determining the overall performance of the composite material, as the propagation of fractures varies based on the characteristics of the binder [31,32].

Table 2 illustrates the binder characteristics. The binders used in the FRP composite materials were divided into thermosets and thermoplastics. Because thermoplastics exhibit lower mechanical properties than thermosets, the amount of thermosetting resins used in composite materials was significantly higher than thermoplastic resins [31,32]. With the recent development of resins exhibiting similar or higher mechanical properties compared to thermosetting resins, the proportion of thermoplastic resins in the binders of composite materials has gradually been increasing [31,32]. Thermosetting resins include unsaturated polyester resin, vinyl ester, and epoxy. Unsaturated polyester resin, which is inexpensive and easy to manipulate, is used as a binder in several composite materials; however, epoxy or vinyl esters are primarily used in FRP tendons or reinforcing bars [33].

### 2.2. CFRP Anchorage Systems

**Figure 1:**
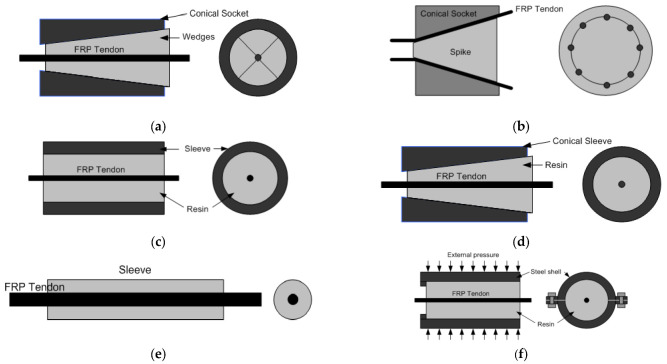
Carbon fiber-reinforced polymer (CFRP) anchorage systems: (**a**) Split-wedge method; (**b**) plug-in-cone method; (**c**) resin–sleeve method; (**d**) resin-potted method; (**e**) soft metal overlay method; and (**f**) split-plate method.

Figure 1 shows the CFRP anchorage systems commonly used by companies [2,34]. Figure 1a illustrates the split-wedge method. Herein, the number of wedges and the geometry of the groove vary depending on the surface and cross-sectional geometry of the FRP. This anchorage system uses mechanical interlocking, wherein the teeth of the wedge anchor the FRP by pressurizing the FRP surface. It transfers the load through the shear pressure (shear gripping type) and comprises a conical socket surrounding the FRP and metal wedges with grooves in the shape of a saw blade. Figure 1b demonstrates the plug-in-cone method, wherein the FRP tendons are anchored between the spike and the conical socket by placing the tendons in the socket and then pushing the spike. For the anchored FRP tendons to exhibit maximum performance, the tendons must be located uniformly around the spike. The mechanism of this system is based on the anchorage system developed for strands by company A in 1936. Satisfactory performance was reported under static and fatigue loads. Figure 1c shows the resin–sleeve method. Herein, the selection of resin is critical because the anchorage performance relies on the bonding between the resin and the FRP tendon. Although using a resin with a small Young’s modulus can decrease the maximum shear stress, a resin with a large Young’s modulus is favorable for long-term creep control. The cylindrical metal or non-metal sleeve is filled with epoxy to anchor the FRP tendon in this system, and the adhesive force generated between the resin and the FRP tendon provides resistance. Figure 1d illustrates the resin-potted method. This anchorage mechanism is a combination of the split-wedge and resin–sleeve methods. The compressive force is applied to the FRP tendon by the split-wedge, strengthening the adhesive force between the resin and the FRP tendon. However, this method has drawbacks such as the reduction in adhesive force owing to moisture, sensitivity to the temperature load, and creep deformation. Figure 1e demonstrates the soft metal overlay method. In this anchorage system, the hollow metal sleeve is installed on the FRP tendon through swaging, similar to the strand swage sleeve. Aluminum or soft steel is used as a material for the sleeve. Overseas, company B use this system for product L, and company C use this metal sleeve anchorage system for CFRP tendons. Figure 1f shows the split-plate method. This anchorage system increases the adhesive force between the resin and the FRP tendon by anchoring the tendon in the resin, installing a plate or a cylinder outside the resin, and applying external force using devices such as bolts. However, slip may be generated in this system.

### 2.3. Test Methods and Results of Single-Anchorage Systems

#### 2.3.1. Specimens of Single-Anchorage System Test

**Figure 2:**
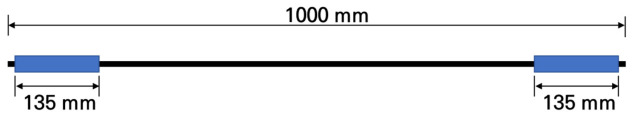
Specification of the CFRP specimen and sleeve.

Figure 2 shows the geometry of a specimen of the CFRP single-anchorage system. The length and diameter of the CFRP used was 1000 mm and 10 mm, respectively. The specimen was fabricated such that 20 to 30 mm could be excluded at both ends to prevent the separation of the compression sleeve owing to the slip generated during the tensile test after pressurization and to ensure symmetry to the highest possible extent between the left and right sides. The specifications of the anchorage sleeve were as follows: the total length was 135 mm, and the total height of the cross-section was 25.9 mm. The geometry of the sleeve maintained a left–right symmetry to ensure that it is not limited by the direction when applied to actual construction sites.

#### 2.3.2. Manufacturing Process of Single-Anchorage System Test Specimens

**Figure 3:**
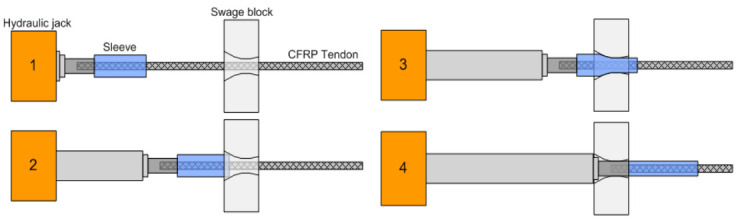
Sleeve pressurization process.

Figure 3 illustrates the process of sleeve pressurization, which is sequentially performed as follows: initially, the steel sleeve was positioned between the swage block and the hydraulic cylinder. The CFRP cable was positioned in a straight line with the hydraulic cylinder after penetrating the swage block and steel sleeve. Subsequently, the hydraulic cylinder performed pressurization while pushing the steel sleeve toward the swage block. Finally, the change in the length of the pressurized steel sleeve was examined.

#### 2.3.3. Single-Anchorage System Tensile Strength Method

**Figure 4:**
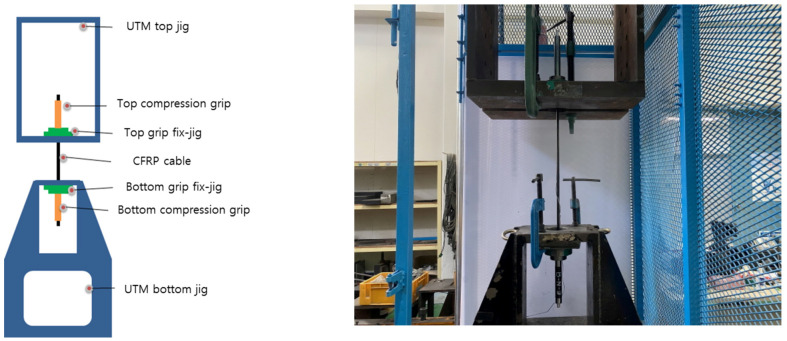
Single-anchorage system tensile test method.

Table 3 and Table 4 and Figure 4 illustrate the single-anchorage system tensile test method. A TML strain gauge (Tokyo Sokki Kenkyujo Co., Ltd., Tokyo, Japan) was attached at the center of the CFRP cable. The major specifications of the strain sensor were as follows: the length and width of the sensing sheet were 5 and 1.5 mm, respectively; the length and width of the base were 10 and 3 mm, respectively; the resistance value was 120. The specimen direction was fixed with the top and bottom jigs, and the specimen was placed using the top and bottom compression grips. The fixed single-anchorage system was tested using a 1000 kN universal testing machine (UTM). Displacement control was performed with a 5 mm/min loading rate, and the test was conducted until the specimen fractured. The specimen was then measured using a static electrical resistance TDS-530 data logger (Tokyo Sokki Kenkyujo Co., Ltd., Tokyo, Japan), which is the most commonly used measuring instrument for structures.

#### 2.3.4. Results of the Single-Anchorage System Test

**Figure 5:**
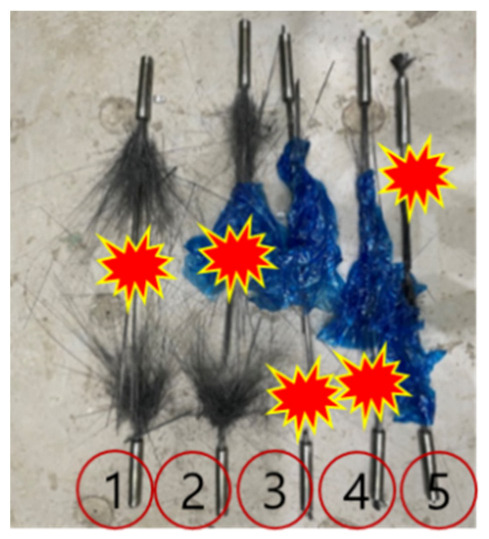
Location of fractures in different specimens.

**Figure 6:**
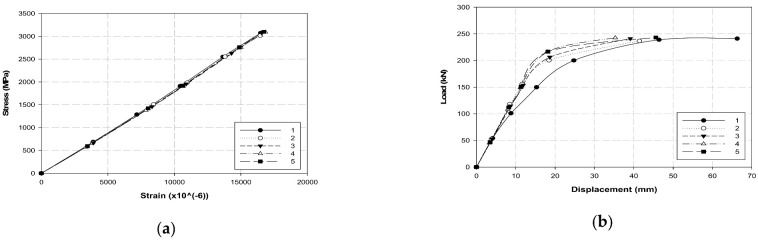
Test results of the single-anchorage system: (**a**) stress and strain and (**b**) load–displacement.

Table 5 and Figure 5 and Figure 6 present the test results of the single-anchorage system and fracture geometry. The average maximum load, tensile strength, and Young’s modulus were 241 kN, 3071 MPa, and 185 GPa, respectively, while their minimum values were 97.5, 97.2, and 96.8% of the maximum values, respectively. This result confirmed that most of the specimens exhibited similar results. A flower-shaped fracture occurred at the center of specimens 1 and 2, whereas a fracture occurred at the inner end of the single-anchorage system in specimens 3 to 5. Furthermore, only the displacement increased when the load approached the maximum value, and a fracture occurred after a certain period (Figure 6b). This phenomenon was caused by the occurrence of slip at the interface between the CFRP cable and the sleeve. Therefore, a larger load could be anticipated without the slip generation. Additional tests were conducted considering different variables to increase the adhesive force.

## 3. Results and Discussion

### 3.1. Performance Improvement of Single-Anchorage System Compression

#### 3.1.1. CFRP Cable Compression Variable Conditions

**Figure 7:**
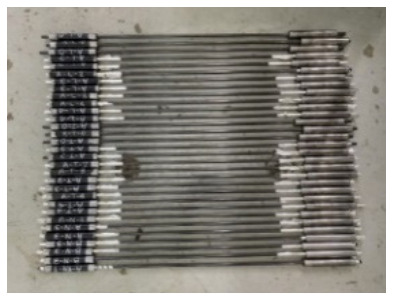
Additional specimens.

Table 6 summarizes the different variables used to increase the adhesive force of the CFRP cable in the single-anchorage system and obtain the maximum load performance; sleeve manufacturers A and B were considered for variable conditions. The surface treatment direction at the interface between the CFRP cable and the sleeve was divided into transverse and longitudinal directions. One to four insert layers between the sleeve and the CFRP cable were set as variables, and three specimens were fabricated for each variable. Furthermore, a variable of a diagonally placed insert was added and compared. Figure 7 demonstrates the specimens prepared according to the variables. The test conducted was identical to the one illustrated in Figure 4.

#### 3.1.2. Test Results

**Figure 8:**
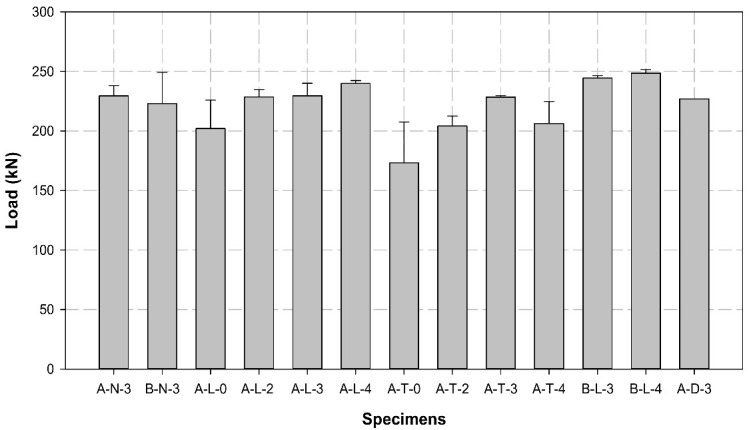
Comparison of the average maximum load of different specimens.

**Figure 9:**
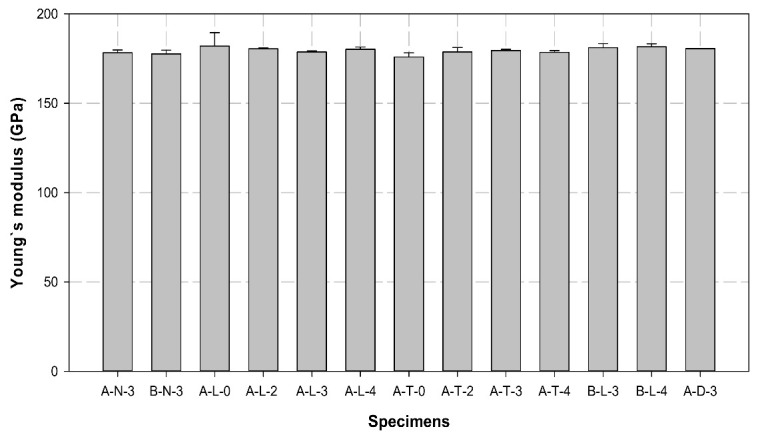
Comparison of the average values of Young’s modulus of different specimens.

**Figure 10:**
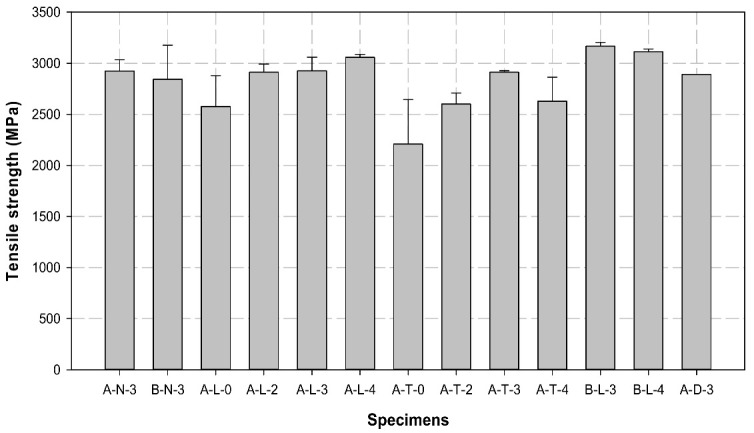
Comparison of the average values of the tensile strength of different specimens.

**Figure 11:**
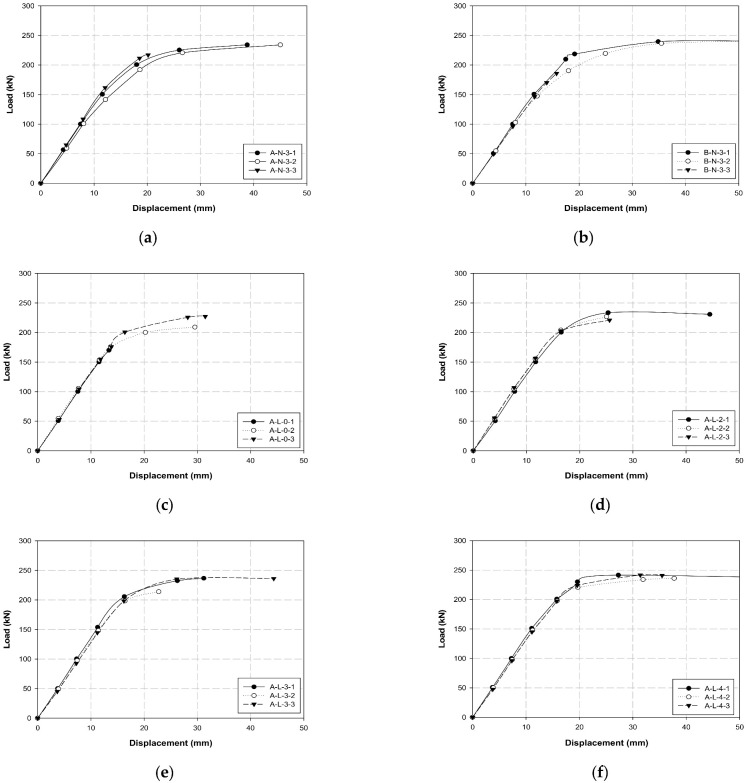

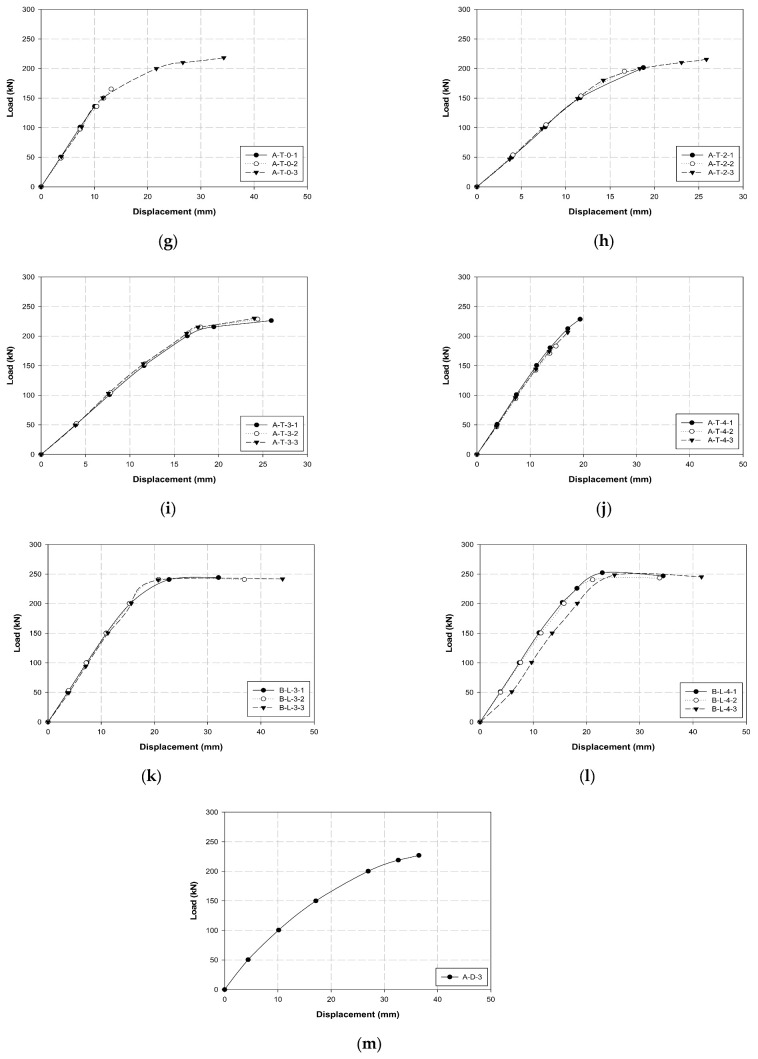
Compression performance improvement load–displacement graphs: (**a**) A-N-3; (**b**) B-N-3; (**c**) A-L-0; (**d**) A-L-2; (**e**) A-L-3; (**f**) A-L-4; (**g**) A-T-0; (**h**) A-T-2; (**i**) A-T-3; (**j**) A-T-4; (**k**) B-L-3; (**l**) B-L-4; and (**m**) A-D-3.

Table 7 lists the maximum load, tensile strength, and Young’s modulus obtained for all specimens. Figure 8, Figure 9 and Figure 10 illustrates the average maximum load, Young’s modulus, and tensile strength of the three specimens for each variable, respectively. Figure 11 shows the load–displacement curves used to analyze the slip and behavior of the specimens.

The average maximum loads for each variable were compared, as shown in Table 3 and Figure 8. The maximum loads of A-N-3 and B-N-3, the control specimens without surface treatment, had a 2% difference of 229.5 and 223.1 kN, respectively.

In the case of A-L variables, the specimens characterized by sleeve manufacturer A and longitudinal CFRP surface treatment exhibited an increase of more than 20% of the maximum load with an increase in the number of insert layers. The maximum load increased by more than 10% with two or three insert layers compared to that of the zero-layer-specimen. The maximum load increased by more than 20% when the number of insert layers was four. Compared to the control specimen A-N-3, A-L-0 with longitudinal surface treatment and no insert layer exhibited an approximate 10% reduction in the maximum load. Additionally, A-L-2 and A-L-3 exhibited similar loads, and A-L-4 exhibited an approximate 10% improvement in the maximum load. Based on these results, we determined that the maximum load improves by approximately 20% when the specimens undergo longitudinal surface treatment and contain a higher number of insert layers.

In terms of A-T variables, the specimens characterized by sleeve manufacturer A and transverse CFRP surface treatment exhibited an increase of more than 24% in the maximum load with an increase in the number of insert layers. However, the load for four layers decreased by more than 10% compared to that for three layers. The maximum load for specimens with two, three, and four insert layers increased by more than 15, 25, and 15%, respectively, compared to the zero-layer-specimen. However, all A-T specimens exhibited lower maximum loads when compared to the control specimen A-N-3, except for A-T-3, which demonstrated a maximum load similar to that of A-N-3. Furthermore, the loads of A-T-0, A-T-2, and A-T-4 decreased by approximately 25, 10, and 10%, respectively, compared to that of the control specimen.

When the A-L and A-T specimens were compared, the CFRP cable with the longitudinal surface treatment exhibited a higher load than that with the transverse surface treatment in all cases. In particular, the load was 17, 12, 1, and 16% higher when 0, 2, 3, and 4 layers were compared, respectively. In the case of the sleeve obtained from company A, specimen A-L-4 with longitudinal surface treatment and four insert layers exhibited the highest performance.

Among the B-L variables, B-L-4 exhibited a slightly higher load of 2% than B-L-3. Additionally, the load was approximately 10% higher than that of the control specimen B-N-3. In contrast to the A-L variables, the loads of B-L-3 and B-L-4 were 10 and 5% higher compared to those of A-L-3 and A-L-4, respectively. Furthermore, the performance of the diagonally reinforced A-D-3 was lower than that of the A-L and B-L specimens.

Finally, the average loads of all specimens were compared. B-L-4 exhibited the highest average load of 248.7 kN, wherein the maximum load of the individual specimen B-L-4-1 was increased by more than 5% up to 252.4 kN. This result indicates that company B, longitudinal surface treatment, and four insert layers are required to increase the adhesive force between the CFRP cable and the compression sleeve. Furthermore, the longitudinal surface treatment exhibited higher performance than the transverse surface treatment because the direction of the transverse surface treatment was perpendicular to that of the cable fibers, which resulted in the breakage of fibers and thereby failure in the performance.

The tensile strength was derived from the maximum load and tended to be similar to the tensile load. The tensile strength of all A-L and A-T variables, except specimen A-T-4, increased with an increase in the number of insert layers. Although the tensile strength of specimen A-L-4 was higher than that of the control specimen, the performance of A-T-4 was slightly low. The B-L specimens exhibited the highest tensile strength among all specimens.

The Young’s modulus of control specimens was approximately 178 GPa, while this varied from 180 (increased by more than 1%) to 182 GPa (increased by more than 2%) in most of the A-L and B-L variables. However, the A-T variables exhibited Young’s modulus of 178 GPa. In comparison with the control specimens, Young’s modulus was 1 to 4% higher in all specimens that included longitudinal surface treatment and insert layers, regardless of the sleeve manufacturer.

Figure 11 shows the load–displacement curves for each variable, wherein the slip generation between the CFRP cable and the sleeve in each specimen can be confirmed. A curve was observed in both control specimens A-N and B-N rather than a straight line from 150 kN to the maximum load before the slip generation. Although slip occurred between the maximum load and the fractured section, displacements of 30 to 45 mm and 30 to 50 mm were observed in A-N and B-N, respectively. The slip generated was higher for company B specimens than those of company A. Compared with the control specimen, A-L specimens exhibited a higher maximum load with an increase in the number of insert layers. Additionally, the slip was insignificant, and linearity was maintained. However, approximately 10% more slip was observed from the maximum load to the fractured section compared to the control specimen. Furthermore, the A-T specimens exhibited significant brittle failure behavior as the number of insert layers increased. Although these specimens reached the maximum load without slip, a fracture occurred under the maximum load without slip as the number of layers increased. However, the maximum load was lower than that of the control specimen. Finally, the B-L (B-L-3 and B-L-4) specimens exhibited a higher maximum load than the control, A-L, and A-T specimens, and formed a considerably accurate linear section without slip generation up to the maximum load. Additionally, we confirmed that the slip from the maximum load to the fractured section was significantly less than that observed in the control and A-L specimens. A comparison of all conditions, including the maximum load, tensile strength, Young’s modulus, and slip generation revealed that the B-L-4 specimen with the largest number of insert layers among the B-L specimens exhibited the best performance. Therefore, specimen B-L-4 was used in the performance test for the three- and seven-multi-anchorage systems.

### 3.2. Test Details and Results

#### 3.2.1. Multi-Anchorage System Details

**Figure 12:**
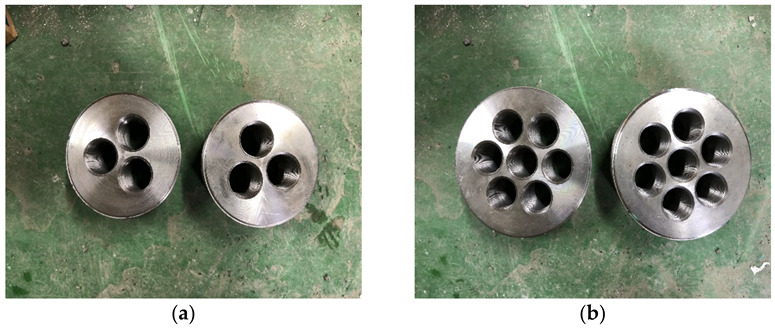

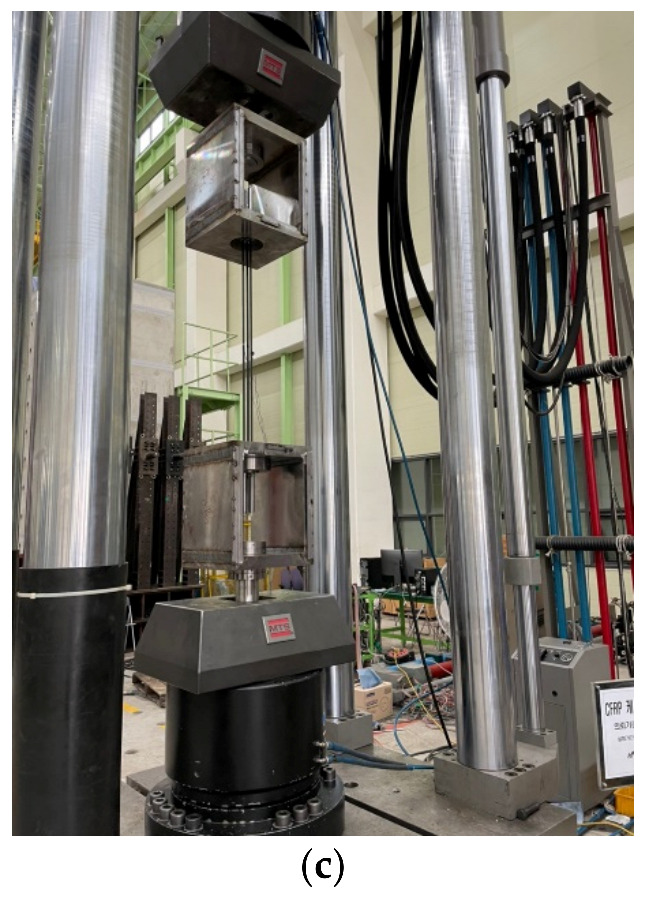
Anchor heads used for (**a**) three-, (**b**) seven-multi-anchorage systems, and (**c**) experimental method.

Figure 12 shows the anchor heads attached to the anchorage systems. Based on the single-anchorage system results summarized in Section 3.1, the B-L-4 specimen was used, and the identical manufacturing process was adopted. The anchor heads were attached by inserting bolts during the manufacturing of the single-anchorage system. Subsequently, the single-anchorage system was placed in the three- and seven-multi anchor heads and fixed using bolts. We prepared three specimens each for three- and seven-multi-anchorage systems. The tensile test was conducted using a 5000 kN UTM, as shown in Figure 4.

#### 3.2.2. Multi-Anchorage System Test Results

**Figure 13:**
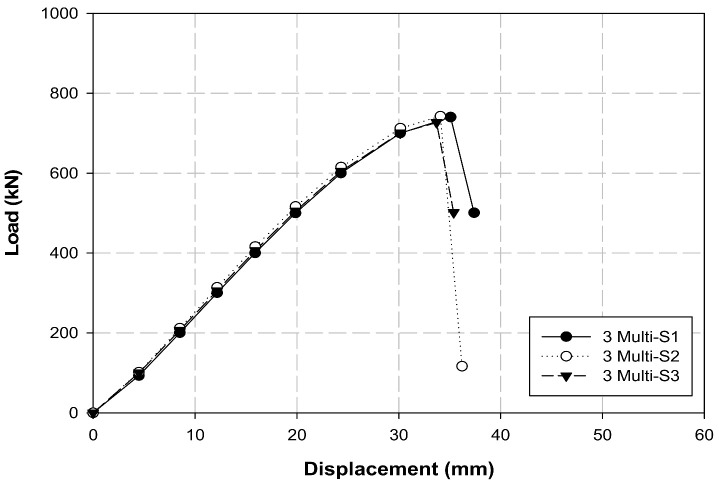
Load–displacement graph of the three-multi-anchorage system.

Table 8 and Figure 13 present the tensile test results of the three-multi-anchorage system. The average maximum load and tensile strength were 736. 7 kN and 9385.1 MPa, respectively, and all three specimens exhibited similar tendencies. The maximum load and tensile strength obtained were 245.1 kN and 3128.4 MPa, respectively, when the average maximum load and tensile strength of the three-multi-anchorage system were calculated with respect to the three-multi-anchorage system. This performance is approximately 100% satisfactory compared with the performance of the B-L-4 single-anchorage system (Section 3.1).

**Figure 14:**
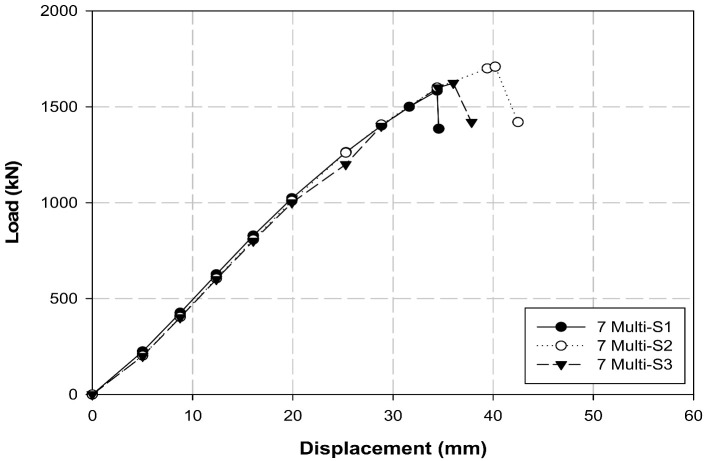
Load–displacement graph of the seven-multi-anchorage system.

Table 9 and Figure 14 present the tensile test results of the seven-multi-anchorage system. The average maximum load and tensile strength were 1638.7 kN and 20,875.6 MPa, respectively. However, approximately 5 to 10% differences in load and tensile strength were observed when the three specimens were compared. The obtained maximum load and tensile strength were 234.1 kN and 2982.2 MPa, respectively, when the average maximum load and tensile strength of the seven-multi-anchorage system were calculated with respect to the one-multi-anchorage system. This performance is approximately 95% satisfactory when compared to the performance of the B-L-4 single-anchorage system. Therefore, we concluded that both three- and seven-multi-anchorage systems perform remarkably. However, although most of the cables in the three-multi-anchorage system fractured simultaneously during the tensile test, the seven cables in the seven-multi-anchorage system tended to fracture at different times. Therefore, considering that the simultaneous fracture of the cables is induced by precise construction of the three- and seven-multi-anchorage systems, the performance can be further improved compared to the single-anchorage system.

## 4. Conclusions

In this study, the bonding performance between the CFRP cable and the sleeve, which served as the anchorage equipment, was improved by performing multiple variable tests. The optimized single-anchorage system was derived based on these tests, and the system was then applied to the three- and seven-multi-anchorage systems. The conclusions of the study can be summarized as follows:

In the case of A-L variables, A-L-4 exhibited an approximately 10% improvement in the maximum load, whereas the load generally decreased by 10 to 25% in the A-T variables. The two, three, and four layers increased the maximum load by more than 15, 25, and 15%, respectively, compared to the zero-layer specimen. The A-L and A-T specimens were compared, and the CFRP cable with the longitudinal surface treatment exhibited a higher load than that with the transverse surface treatment in all cases. In particular, the load was 17, 12, 1, and 16% higher when 0, 2, 3, and 4 layers were compared, respectively. In comparison with the A-L variables, loads of B-L-3 and B-L-4 were 10 and 5% higher than that of A-L-3 and A-L-4, respectively; B-L-4 exhibited a slightly 2% higher load than B-L-3. When the behavior (slip) of the B-L-4 specimen was compared with that of other specimens, it was identified as the optimal single-anchorage system owing to its linearity up to the maximum load and the least slip from the maximum load to the fractured section. This result indicates that company B, longitudinal surface treatment, and four insert layers are required to increase the adhesive force between the CFRP cable and the compression sleeve. Furthermore, the longitudinal surface treatment exhibited higher performance than the transverse surface treatment; the transverse surface treatment failed to perform appropriately because its direction was perpendicular to the cable fibers, which resulted in the breakage of fibers. The tensile test results for the three- and seven-multi-anchorage systems indicated that the maximum loads were 736.7 and 1638.7 kN and the tensile strengths were 9385.1 and 20,875.6 MPa, respectively. Furthermore, the three- and seven-multi-anchorage systems exhibited maximum loads of 245.1 and 234.1 kN and tensile strengths of 3128.4 and 2982.2 MPa, respectively, in terms of the seven-multi-anchorage system. These results verify that the three- and seven-multi-anchorage systems satisfied 100 and 95% tensile performances, respectively, compared to the B-L-4 specimen. Therefore, improved results can be obtained when the construction problems of multi-anchorage systems are addressed in the future. Although multiple variable tests were conducted in this study to increase the maximum load and improve the bonding performance between the CFRP cable and compression sleeve, the anchorage performance may vary depending on the type of fiber, such as GFRPs and AFRPs, and the cable type. Therefore, in the future, we intend to perform variable tests with different materials to verify the performance using diverse types of anchorage systems.

## Figures and Tables

**Table 1:** Characteristics of the FRP fibers.

Category			Specific Gravity	Tensile Strength (MPa)	Young’s Modulus (GPa)	Elongation (%)	Coefficient of Thermal Expansion (×10^−6^/°C)	Poisson’s Ratio
Glass fiber		E	2.54	600–3500	5–74	4.8	5.0	0.2
		S	2.49	4900	87	5.6	2.9	0.22
		AR	2.78	1800–3500	6–70	2.0–3.0	N/A	N/A
Carbon	Pan	High-strength	1.76	3500	200–240	1.3–1.8	−1.2~−0.1 (Longitudinal)	–0.2
		High elasticity	1.76	2500–4000	350–650	0.4–0.8	7~12 (Lateral)	−0.2
	Pitch	General	2.00	780–1000	38–40	2.1–2.5	−1.6~−0.8 (Longitudinal)	N/A
		High elasticity	2.00	3000–3500	400–800	0.4–1.5	−1.6~−0.8 (Longitudinal)	N/A
Aramid fiber		Kevlar 29	1.45	3620	82.7	4.4	N/A	0.35
		Kevlar 49	1.45	2800	130	2.3	−2.0 (Longitudinal), 59 (Lateral)	0.35
		Kevlar 129	1.45	4210	110	-	N/A	0.35
		Kevlar 149	1.45	3450	172–179	1.9	N/A	0.35
		Twaron	1.45	2800	130	2.3	−2.0 (Longitudinal), 59 (Lateral)	0.35
		Technora	1.39	3500	74	4.6	N/A	0.35

**Table 2:** Characteristics of the binders.

Category		Specific Gravity	Tensile Strength (MPa)	Young’s Modulus (GPa)	Coefficient of Thermal Expansion (×10^−6^/°C)
Thermoset	Polyester	1.10–1.40	45–90	2.5–4.0	100–110
	Vinylester	1.12–13.3	73–81	3.0–3.35	100–110
	Epoxy	1.20–1.30	90–110	3.0–7.0	45–65
	Phenol	1.5–1.75	45–59	5.5–8.3	30–45
Thermoplastic	PVC	1.37	58	2.4–2.8	50
	ABS	1.05	17–62	0.69–2.82	60–130
	Nylon	1.13–1.15	48–83	1.03–2.76	80–150

**Table 3:** Capacity of a 1000 kN universal testing machine (UTM).

	Specifications	Details
1	Model name	1000 kN Dynamic UTM
2	Experimental capacity	Loading rage: +1000 to −1000 kN
		Displacement range: 0–500 mm

**Table 4:** Capacity of a TDS-530 data logger.

	Specifications	Details
1	Model name	1000 kN Dynamic UTM
2	Measurement point channel	Basic channel: 30 channel Switch box (added): 1000 channel
3	Measurement target	Strain, DC voltage, Thermocouple
4	Scanning time	IHW-50G: 0.4 s/1000 points (1 s/point)
5	Dimensions	320 (W) × 130 (H) × 440 (D) mm
6	Weight	Approximately 8 kg
7	Power supply	AC 85-250 V 50/60 Hz 114 VA MAX

**Table 5:** Tensile test results of the single-anchorage system.

Specimens (EA)	CFRP Cable			
	Maximum Load (kN)	Tensile Strength (MPa)	Young’s Modulus (GPa)	Fracture Location
1	241	3075	189	Center
2	237	3015	183	Center
3	241	3072	183	End
4	243	3098	184	End
5	243	3099	187	End
Average	241	3071	185	-

**Table 6:** Optimization of variables for performance improvement.

Specimens	Sleeve Manufacturer	CFRP Surface Treatment Direction	Number of Insert Layers	Specimens (EA)
A-N-3	A	-	3	3
B-N-3	B	-	3	3
A-L-0	A	Longitudinal	0	3
A-L-2	A	Longitudinal	2	3
A-L-3	A	Longitudinal	3	3
A-L-4	A	Longitudinal	4	3
A-T-0	A	Transverse	0	3
A-T-2	A	Transverse	2	3
A-T-3	A	Transverse	3	3
A-T-4	A	Transverse	4	3
B-L-3	B	Longitudinal	3	3
B-L-4	B	Longitudinal	4	3
A-D-3	A	Longitudinal	3	1

**Table 7:** Test results for performance improvement.

Specimens	Maximum Load (kN)	Tensile Strength (MPa)	Young’s Modulus (GPa)
A-N-3-1	236.4	3011	180.2
A-N-3-2	234.9	2992	178.3
A-N-3-3	217.2	2767	176.5
A-N-3 Avg.	229.5	2924	178.3
B-N-3-1	242.4	3088	177.8
B-N-3-2	240.9	3069	180.2
B-N-3-3	185.9	2368	174.9
B-N-3 Avg.	223.1	2842	177.6
A-L-0-1	170.2	2168	192.7
A-L-0-2	209.3	2666	177.1
A-L-0-3	227.0	2892	176.4
A-L-0 Avg.	202.2	2576	182.0
A-L-2-1	237.0	3019	179.8
A-L-2-2	226.9	2890	181.1
A-L-2-3	222.2	2831	180.4
A-L-2 Avg.	228.7	2913	180.5
A-L-3-1	236.8	3017	179.6
A-L-3-2	214.9	2738	177.9
A-L-3-3	237.3	3023	178.8
A-L-3 Avg.	229.7	2926	178.7
A-L-4-1	241.7	3079	180.4
A-L-4-2	236.6	3014	181.7
A-L-4-3	241.7	3079	178.5
A-L-4 Avg.	240.0	3057	180.2
A-T-0-1	136.2	1735	173.0
A-T-0-2	165.4	2107	175.8
A-T-0-3	218.8	2787	178.9
A-T-0 Avg.	173.4	2209	175.9
A-T-2-1	201.7	2569	176.8
A-T-2-2	195.3	2488	177.1
A-T-2-3	215.5	2745	182.3
A-T-2 Avg.	204.2	2601	178.7
A-T-3-1	227.0	2892	179.4
A-T-3-2	228.7	2913	179.0
A-T-3-3	230.1	2931	180.4
A-T-3 Avg.	228.6	2912	179.6
A-T-4-1	228.5	2911	179.9
A-T-4-2	183.2	2334	178.1
A-T-4-3	207.3	2641	177.6
A-T-4 Avg.	206.3	2628	178.5
B-L-3-1	246	3134	179.2
B-L-3-2	241.4	3075	182.5
B-L-3-3	245.9	3132	183.0
B-L-3 Avg.	244.4	3113	181.6
B-L-4-1	252.4	3215	181.6
B-L-4-2	245.2	3124	178.3
B-L-4-3	248.6	3167	183.6
B-L-4 Avg.	248.7	3168	181.2
A-D-3	227.0	2892	180.6

**Table 8:** Three-multi-anchorage system test results.

Specimens	Maximum Load (kN)	Tensile Strength (MPa)	Displacement (mm)
B-L-4	248.7	3168	36.6
3-multi S1 (1EA)	740.4	9431.8	46.2
3-multi S2 (1EA)	742.9	9463.7	36.2
3-multi S3 (1EA)	726.9	9259.9	35.6
3-multi-S Avg. (1 single)	736.7 (245.1)	9385.1 (3128.4)	39.3 (13.1)

**Table 9:** Seven-multi-anchorage system test results.

Specimens	Maximum Load (kN)	Tensile Strength (MPa)	Displacement (mm)
B-L-4	248.7	3168	36.6
7-multi S1 (1EA)	1583.7	20 174.5	42.0
7-multi S2 (1EA	1708.8	21 768.2	42.5
7-multi S3 (1EA)	1623.7	20 684.1	39.0
7-multi-S Avg. (1 single)	1638.7 (234.1)	20 875.6 (2982.2)	41.2 (5.9)

## Data Availability

The data presented in this study are available upon request from the corresponding author.
